# An audiological profile of a cohort of school-aged children with HIV and AIDS attending an antiretroviral clinic in South Africa

**DOI:** 10.4102/sajcd.v67i1.651

**Published:** 2020-04-20

**Authors:** Vuyelwa Z. Peter, Jessica Paken, Lavanithum Joseph

**Affiliations:** 1 Discipline of Audiology, School of Health Sciences, College of Health Sciences, University of KwaZulu-Natal, Durban, South Africa

## Abstract

**Background:**

Recent estimates reveal that there are approximately 280 000 children between the ages of birth and 14 years who are living with the human immunodeficiency virus (HIV) and acquired immunodeficiency syndrome (AIDS) in South Africa. These children are living with a compromised immune system, are vulnerable to opportunistic infections and subsequent hearing loss. However, there is limited research on the nature and extent of this sensory impairment amongst school-aged children.

**Objective:**

This study aimed to determine an audiological profile of a cohort of school-aged children attending an antiretroviral (ARV) clinic, describing the occurrence of hearing loss and nature in terms of degree, type, configuration and symmetry.

**Methods:**

A non-experimental descriptive exploratory study was conducted, where 30 children aged between 6 and 12 years underwent diagnostic audiological assessments. Audiological procedures included case history, medical record review, otoscopic examination, immittance audiometry, pure-tone audiometry, speech audiometry, distortion product otoacoustic emissions (DPOAEs) and neurological auditory brainstem response (ABR) testing. The results were analysed descriptively using SPSS version 22 software.

**Results:**

The results indicated abnormal otoscopic findings in half the participants, and consequently type C tympanograms were the most common. Of the 28 participants who could be assessed with pure-tone audiometry, 15 (54%) showed a hearing loss. A bilateral rising mild, conductive hearing loss was predominant. Thirteen (43%) of the participants could not be tested using DPOAE because of outer and middle-ear pathology. Neurological ABR testing revealed an abnormality in 18 (60%) of the participants suggesting the sensitivity of the ABR to detect subtle neurological changes.

**Conclusion:**

Half the children in this study showed hearing loss, which has serious implications for the holistic management of the children within the health and educational contexts. Therefore, there is a need for audiological monitoring of children with HIV and AIDS.

**Keywords:**

audiological profile; HIV; AIDS; hearing loss; school children.

## Introduction

The Joint United Nations Programme on human immunodeficiency virus (HIV) and acquired immunodeficiency syndrome (AIDS) estimated that approximately 280 000 children in South Africa were infected with HIV in 2016 (United Nations Acquired Immuno-Deficiency Syndrome [UNAIDS], 2016). Furthermore, they reported that 55% of children living with HIV in South Africa are on antiretroviral (ARV) treatment. The global use of ARV is reported to have cut the number of deaths in half amongst children (aged 0–14 years) between 2010 and 2016, the major reason being increased access to paediatric ARV therapy, especially in Eastern and Southern Africa (UNAIDS, 2017). Thus, the disease and its treatment are of major interest worldwide.

Literature suggests that the HIV virus does not only attack the immune system but may also affect the function of different organs of the body, including the auditory system, which can result in hearing loss. The reported cause of hearing loss in these children can be attributed to otologic infections (Gurney & Murr, 2003), which may give rise to a conductive (CHL), mixed (MXHL) or sensorineural hearing loss (SNHL), as well as central auditory deficits, which could affect language development (Abrams, Moon, Robinson, & Van Dyke, 2006; Lasis, 2005). The hearing loss can thus manifest in young children through failure to attain language developmental milestones on par with other children (Makar, Dhara, Sinha, Chatterjee, & Dutta, 2012). Language is essential for thinking, communication learning, and hearing loss may interfere with successful learning (Donald, Walker, Riordan, Govender, & Wilmshurst, 2012). This may result in poor educational outcomes and future prospects of employment (Feagans, Kipp, & Blood, 1994), with a life-long effect on the quality of life.

A study by Shapiro and Novelli (1998) on children HIV infection indicated that 32 (44.4%) of the participants presented with one or more episodes of middle-ear pathology, which was otitis media. Whilst the results of a pathogen type of immunocompetent children were similar to the general population of children not infected with HIV, the results suggested that the severity of immunosuppression was associated with a higher incidence of middle-ear pathology. Another study by Matas, Leite, Leite-Magliaro and Gonçalves (2006) revealed that children with AIDS had more abnormal results than their counterparts. The authors suggested that AIDS be recommended as a risk factor for peripheral and/or auditory brainstem disorders. It also highlighted the need for regular follow up and monitoring of the peripheral and brainsterm pathways. This is further supported by Palacios et al. (2008) who recommended that children with HIV and AIDS should undergo audiological evaluation at an earlier stage for immediate intervention to minimise the psychosocial impact of hearing loss on their development. Matas, dos Santos Filho, de Juan, Pinto and Gonçlaves (2010) further added that because of the growing incidence of hearing loss in HIV-positive people, audiological as well as electrophysiological investigations of hearing should be conducted to detect the alterations of the peripheral and central auditory pathways as the stages of HIV infection progress to AIDS stage. Thus, it becomes vitally important to conduct hearing assessments on children who appear to be at high risk for hearing loss because of their HIV status. In-depth hearing assessment would shed light on the auditory manifestation of the disease as studies conducted on the adult population report a notable prevalence of sensorineural hearing loss (Khoza & Ross, 2002; Khoza-Shangase, 2010; Makar et al., 2012; North-Matthiassen & Singh, 2006; Paken, 2007). Ototoxic medication in response to treating infections may also contribute to hearing loss; however, such studies are limited in the paediatric population (Khoza-Shangase, 2010; Makar et al., 2012).

Whilst studies have highlighted the need for audiological assessment in the paediatric population, the actual prevalence and nature of hearing loss in this population is still not known, especially in South Africa, as most published research relating to HIV and AIDS and hearing research is focussed on the adult population (Khoza-Shangase, 2010). There are limited published findings in South Africa that report on the auditory status of HIV-infected children, and there is a need for such research to be published (Moragan et al., 2002). This study, therefore, posed the question, what is the audiological profile of children with HIV and AIDS in South Africa? Therefore, this study aimed to determine the audiological profile of a cohort of school-aged children with HIV and AIDS during their medical monitoring at an urban public sector ARV clinic in KwaZulu-Natal (KZN) South Africa.

## Methods

### Study aim

To determine an audiological profile of a cohort of school-aged children with HIV and AIDS at an ARV clinic in KZN.

### Study objective

To describe the occurrence and nature of hearing loss in terms of type, degree, symmetry and configuration.

### Study design

A descriptive exploratory study design with non-probability convenience sampling was used. Convenience sampling was used for the site selection as the primary investigator was employed at the study site during the data collection phase. Participants were recruited and assessed once using a non-probability consecutive sampling technique (Cresswell, 2009). The study was conducted at an ARV clinic in an urban public hospital in Durban, which is a metropolitan hub of KZN.

### Description of study participants

Thirty children were recruited for this study, with 17 (60%) males and 13 (40%) females, and an age range of 6–12 years. Participant’s grades ranged from Grade R to Grade 7. The mean age of the children was 4.3 years. There were 17 children in the foundation phase and 13 participants in the senior phase. Twenty-nine participants (97%) were on ARV treatment and one participant (3%) was ARV naive at the time of testing, as his viral load was below detectable levels. Twenty-three (79%) participants had been on ARV for longer than 5 years, and six (20%) participants have been on ARV for less than 5 years. Twenty-one (72%) of the participants were on regimen two (consisting of zidovudine [AZT] + Epivir [3TC] + Efavirenz [EFV]) whilst eight (27%) participants were on regimen one (Abacavir [ABC] + 3TC + EFV) treatment. Twenty-eight (93%) of the participants had Cluster of differentiation -4 (CD4) cell count category results reordered in their medical during the data collection phase of the study, and it ranged from 1 to 3 as reflected in Table 1. Majority of the participants (89%) were in the moderate category with CD4 cell of between 200 mm^3^ and 500mm^3^.

**TABLE 1:** Cluster of differentiation 4 (CD4) percentage and CD4 count for children according to Word Health Organization 2015 guidelines.

HIV disease category	CD4 percentage range	Percentage of participants (*n* = 28)		CD4 6- to 12-year-old
		*n*	%	
1 No damage	25% or over	1	4	Over 500 mm^3^
2 Moderate	12% – 24%	25	89	200 mm^3^–500 mm^3^
3 Severe	under 15%	2	7	under 200 mm^3^

*Source*: Doharty, M. (2015). *WHO guidelines on the use of CD4, viral load & EID tests for initiation and monitoring of ARV*. Retrieved from https://www.who.int/hiv/amds/102_WHO_Guidelines_on_CD4_and_VL_for_ART_Doherty.pdf

CD4, Cluster of differentiation 4; HIV, human immunodeficiency virus.

### Data collection procedure

The audiological evaluation followed the standard practice for children with a test battery approach and cross-check principle to ensure that results correlate (Jacobson & Jacobson, 2004) using the following seven tests, materials and equipment:

- A case history questionnaire was developed for this study. The questions were developed based on literature recommendations regarding the development of case history questionnaire. The questionnaire consisted of 26 questions, of which 10 were closed-ended and 16 were open-ended questions. This addressed the concern on content validity; the questions were translated into isiZulu and back translated into English. The primary investigator is a first language isiZulu speaker, and there was no need for an interpreter. The caregiver (parents or close relatives) who accompanied the child to the assessment provided the case history details.
- A medical record form was developed for the purpose of this study. It consisted of three sections that used a checklist to capture the required information. Information on the following pertinent areas was included: CD4 cell measures within a 6-month period; viral load measures within 6 months; ARV regimen was recorded for those who had commenced ARV; other prescribed drugs for other medically related conditions; audiological interventions including audiological tests administered; and results and management strategies employed to manage the presenting audiological conditions.
- An otoscopic examination was conducted using a wall-mounted Welch Allyn diagnostic otoscope. The purpose was the identification of any outer ear abnormalities (Gelfand, 2001).
- Immittance audiometry, including tympanometry and acoustic reflex threshold testing, conducted using the Interacoustic AZ 26 middle-ear analyser with a frequency tone of 226 Hz (Gelfand, 2001).
- Distortion product otoacoustic emissions (DPOAEs) were conducted using the GSI Audera DPOAE.
- Pure-tone audiometry, including both air and bone conduction testing, was carried out using the Madsen Otometrics Itera II audiometer. Air conduction pure-tone audiometry was conducted at every octave frequency from 250 Hz to 8000 Hz, with bone conduction audiometry ranging from 250 Hz to 4000 Hz. Normal hearing was defined to be thresholds of less than 15 dB across all the frequencies (Gelfand, 2001).
- Speech audiometry testing included Speech Reception Thresholds (SRT) obtained through live speech testing in a soundproof booth using a digit list by Ramkisson (2002). The digit word list was used for isiZulu-speaking participants, as there was no validated word list during the time of this study. This was to address construct validity concerns. Speech Discrimination Testing (SD) to obtain Word Recognition Scores was conducted using the isiZulu discrimination word list developed by Balkisoon (2001) for isiZulu-speaking children in KZN. The list factored in cultural, linguistic and age appropriateness, using evidence-based principles accounting for reliability and validity concerns. No validated word lists developed for children were available during the time of the study.
- Auditory Brainstem Response (ABR) testing used a Click. Click stimuli with a rarefaction polarity at an intensity of 80dBHL, duration of 0.1 ms and speed of presentation of 11.1 ms stimulation per second were used. Values of absolute latencies for waves I, III and V and interpeak latencies I–III, III–V and I–V were identified. The ABR was obtained using the GSI Audera Auditory Evoked Potentials (AEP) in a sound-treated room.
- Testing was conducted on the same day. Testing was conducted in a soundproof booth and sound-treated rooms to ensure reliable results (Gelfand, 2001). The participants were provided with comfort breaks between the tests to improve the concentration and reliability of the results (Babbie, 2008). Each participant underwent testing for 2 hours. The order of testing assessment followed this sequence: a case history, medical review, otoscopic examination, immittance, pure tone, speech audiometry, DPOAE and ABR with natural sleep. Feedback on the test results was provided in the language of the client and if referrals to other health care professionals were required, an appointment was secured and referral letter issued on the same day.
- All equipment had undergone calibration to ensure reliable results.
- Participants were recruited from the ARV clinic at the selected hospital with the assistance of the clinic nurse during the data collection period.
- Results were classified as either normal or abnormal using audiological normative data such as normal otoscopic examination findings, type A tympanogram, and ipsilateral and contralateral acoustic reflex thresholds between 70 dB and 100 dB SPL. Pure-tone audiometry across all frequencies of less than 15dB and SRT and pure-tone average agreement between 0 dB and 5 dB.
- The results were descriptively analysed and classified as either normal or abnormal. Data were analysed using SPSS version 22 software with the assistance of a statistician. Descriptive results were conducted using percentages, and frequency.
- Ethical and legal considerations were in line with the World Medical Association’s 2008 Helsinki Declaration, which included informed caregiver consent and child assent. The participants’ identities were kept confidential as they were assigned numbers for identification purposes.
- A pilot study was conducted on five participants to establish whether the case history questionnaire questions were clear and to identify ambiguity. The pilot study aimed to assess the rate, ease of recruiting participants and the time to conduct a full audiological test on each participant (Babbie, 2008; Gravett & Foronzo, 2009). The recommendations from the participants of the pilot study were used to guide the data collection process (Shi, 2008). The participants in the pilot study were not included in the main study. The pilot study assisted to address concerns on process issues such as participant recruitment and legible criteria. In addition, it addressed resource concerns such as time for testing, equipment reliability, referral pathways and if the testing procedure resulted in the correct identification of hearing loss (Thabane et al., 2010).
- Construct validity was accounted for by a pilot study, and the use of valid audiological and calibrated equipment. Reliability was ensured by providing short breaks between tests, and ABR result interpretation was confirmed by an expert in the field and by adhering to a data collection schedule (Creswell, 2009).
- Children identified to have a hearing loss and other medical conditions were referred to the relevant health care professionals within the facility.

### Ethical consideration

Ethical approval was obtained from the University of KwaZulu-Natal Biomedical Research and Ethics Committee (Ethical Clearance No. BE051/13), the Provincial KwaZulu-Natal Department of Health Research Office and the Chief Executive Officer at the study site. Written informed consent was obtained from the caregiver, and written assent was obtained from each child participant in the study (Greig, Taylor, & MacKay, 2007).

## Results

### Case history interview results

Half (15; 50%) of the participants reported delays in speech-language developmental milestones, and 14 (46%) participants had other coexisting medical conditions that included tuberculosis, epilepsy and encephalopathy. Thirteen (43%) participants were suspected of hearing loss by the caregivers, and 24 (80%) participants had never had an audiological assessment although they had reported ear pathology to other health care professionals. Concerning educational history, 14 (47%) of the participants had repeated a grade whilst 15 (50%) reported not being able to cope academically in the current grade. Audiological results are presented below in terms of right and left ears.

### Otoscopic examination results

Observations revealed the presence of occlusive impacted wax (eight right and six left ears), discharge (two left and two right ears) and perforated tympanic membranes (four right and five left ears).

### Immittance audiometry

Type A tympanogram, suggesting normal middle-ear function, was evident in the right ear of 17 participants (68%) and the left ear of 18 participants (75%), as indicated in Table 2. As to be expected, results of ipsilateral and contralateral reflexes were significantly influenced by the status of the middle ear. Five participants could not be tested because of the presence of discharge. Abnormal results included no responses and elevated thresholds. Thus, 12 (40%) of the participants had abnormal immittance results.

**TABLE 2:** Immittance audiometry results.

Immittance (*n* = 30)	Right ear		Left ear	
	*n*	%	*n*	%
**Tympanometry**				
Type A	17	68	18	75
Type B	2	8	3	12.5
Type C	5	20	3	12.5
Type Ad	1	4	0	-
Subtotal	25	100	24	100
Could not test	5	16.6	6	20
**Total**	**30**	**-**	**30**	**-**
**Acoustic reflexes**				
Normal ipsilateral acoustic reflexes	17	68	18	72
Abnormal ipsilateral acoustic reflexes	8	32	7	28
Subtotal	25	100	25	100
Could not test	5	16	5	16
**Total**	**30**	**-**	**30**	**-**
Normal contralateral acoustic reflexes	13	52	13	52
Abnormal contralateral acoustic reflexes	12	48	12	48
Subtotal	25	100	25	100
Could not test	5	20	5	17
Total	30	-	30	-

### Pure-tone audiometry

**FIGURE 1:**
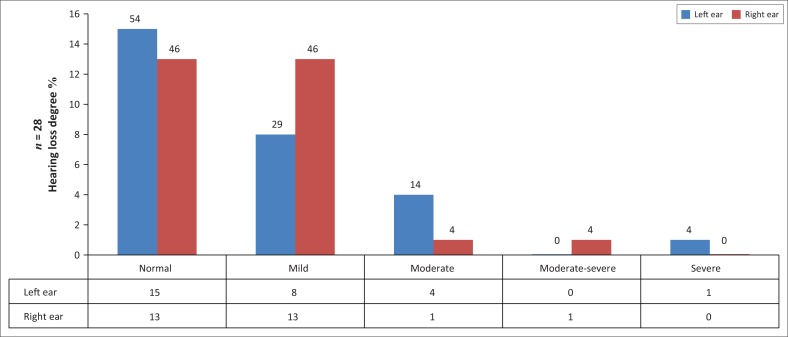
Hearing loss degree.

**FIGURE 2:**
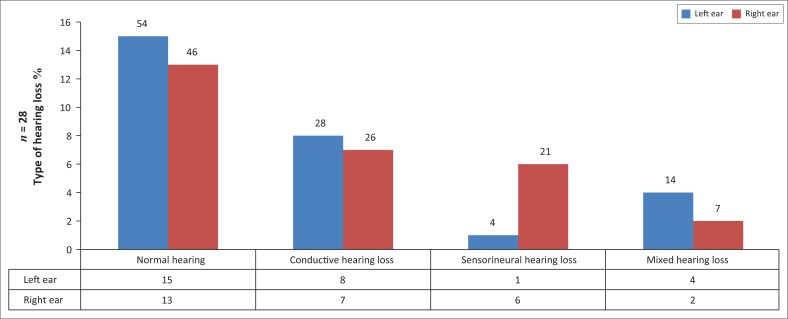
Types of hearing loss.

Pure-tone audiometry was conducted on 28 participants (*n* = 28), as two children could not be conditioned for testing. Of the 28 participants assessed, hearing loss was observed in the right ear of 15 (54%) and left ear of 13 (46%) participants. Of the 16 (57%) participants who presented with a hearing loss in either one or both ears, four (25%) presented with a unilateral hearing loss, whilst the majority 12 (75%) had bilateral hearing loss, with six (38%) having symmetrical hearing loss. Only 12 (43%) of those tested presented with hearing within normal limits. Normal hearing was classified with thresholds better than 15 dB across all the frequencies (Gelfand, 2001). Mild hearing loss was the most common degree of hearing loss observed in 12 (42%) participants, as depicted in Figure 1. Conductive hearing loss was the predominant type of hearing loss observed in eight (29%) participants, as depicted in Figure 2.

The most common configuration was a rising pattern, which is commonly associated with conductive hearing loss.

### Speech audiometry results

Speech audiometry results are reflected in Table 3. The majority of participants obtained good SRT-PTA correlation, indicating good pure-tone test reliability. Good correlation was considered when the SRT-PTA correlation was less than 5 dB, fair correlation between 6 dB and 9 dB and poor correlation greater than 10 dB (Gelfand, 2001). Ninety-six per cent of the participants obtained 90% – 100% speech discrimination, which is in keeping with the pure-tone audiometry results and conductive hearing loss. Word Recognition Scores of more than 88% are considered good (Gelfand, 2001).

**TABLE 3:** Result of speech audiometry testing (*n* = 28).

Pure-tone testing reliability	Right ear		Left ear	
	*n*	%	*n*	%
Good SRT-PTA	23	82	25	89
Fair SRT-PTA	2	7	0	-
Poor SRT-PTA	3	11	3	11
**Word recognition scores**				
90% – 100%	27	96	27	96
< 50%	1	4	1	4

### Electrophysiology

Twelve (40%) of the participants were not assessed using DPOAE because the presence of outer- and middle-ear pathology. Majority the sample (83%) had a pass result for the right ear, with 12 (70%) having a pass in the left ear. Half of the sample (15; 50%) had abnormal absolute latency for the right ear and 10 (33%) for the left ear (Tables 4 and 5).

**TABLE 4:** Distortion product otoacoustic emission distortion product otoacoustic emissions results.

DOAE result (*n* = 30)	Right ear		Left ear	
	*n*	%	*n*	%
Pass	15	83.3	12	70.6
Refer	3	16.6	5	29.4
Subtotal	18	100	17	100
Could not test	12	40	13	43
**Total**	**30**	**-**	**30**	**-**

DOAE, distortion product otoacoustic emissions.

**TABLE 5:** Neurological auditory brainstem response ABR results.

ABR result (*n* = 30)	Right ear (*n* = 30)		Left ear (*n* = 30)	
	*n*	%	*n*	%
**Absolute latency I**
Normal	20	67	15	50
Early	2	7	3	10
Delayed	4	13	7	23
Absent	4	13	5	17
**Total**	**30**	**-**	**30**	**-**
**Absolute latency III**				
Normal	20	67	14	46
Early	4	13	2	7
Delayed	2	7	9	30
Absent	4	13	5	17
**Total**	**30**	**-**	**30**	**-**
**Absolute latency V**				
Normal	20	67	16	53
Early	5	17	2	7
Delayed	1	3	7	23
Absent	4	13	5	17
**Total**	**30**	**-**	**30**	**-**
**Inter-peak latency I–III**				
Normal	19	64	16	53
Early	6	20	6	20
Delayed	1	3	3	10
Absent	4	13	5	17
**Total**	**30**	**-**	**30**	**-**
**Interpeak latency III–V**				
Normal	18	61	19	64
Early	4	13	4	13
Delayed	4	13	2	6
Absent	4	13	5	17
**Total**	**30**	**-**	**30**	**-**
**Interpeak latency I–V**				
Normal	17	57	16	53
Early	9	30	6	20
Delayed	0	-	3	10
Absent	4	13	5	17
**Total**	**30**	**-**	**30**	**-**

## Discussion

Almost half the children in this study had some degree of hearing loss. These findings are higher than the prevalence of 32% for the age group of 7–12 years reported by Matas et al. (2010). Ensink & Kupper (2017), in their review of studies on hearing loss in people infected with HIV, reported a prevalence range of 22% – 13% in children. Whilst this finding may be attributed to the small sample size in the current study, it is in line with reports that South Africa has a high prevalence of children with HIV and AIDS globally. However, the occurrence of hearing loss in this population has not been adequately reported in spite of the high HIV and AIDS prevalence (UNICEF, 2012). One can conclude that there are varying prevalence rates globally related to hearing loss associated with the HIV and AIDS population. Therefore, regular audiological monitoring is warranted, and the researchers concur with the view of Palacios et al. (2008) supporting this. Regular monitoring and documentation will assist in understanding the audiological manifestations of hearing loss in children infected with HIV, as there are limited studies that have reported on the prevalence of hearing loss in this population.

In this present study, the degree of hearing loss varied across the participants, with the majority of the participants having mild hearing loss, that is, eight (29%) of right ears and 13 (46%) of left ears. These results agree with the findings of De Lange (2007) and Khoza and Ross (2002) who found mild hearing loss to be the predominant degree of hearing impairment in the adult population with HIV and AIDS. However, in children, a mild hearing loss is usually not detected easily, and one of the symptoms is auditory fatigue in school because of the extra effort required to listen (Paluski & Kaderavek, 2002). Early-onset of any hearing loss could result in reduced processing at the lower brainstem and cortical levels in subsequent years (Maruthy & Mannarukrishniah, 2008). Auditory processing deficits may manifest as poor concentration, auditory recall, auditory analysis and synthesis. These aspects are, however, essential for reading and writing abilities (Feagans et al., 1994). Therefore, language and auditory processing deficits, resulting from a hearing loss, can interfere with the successful acquisition of literacy skills (Carney & Moeller, 1998). Children with reading fluency problems associated with hearing loss also present with difficulties in reading comprehension (Chandrasekhar et al., 2000). It is, therefore, imperative that early identification of hearing loss be established to improve the academic performance and vocational potential of each child (Paluski & Kaderavek, 2002). The significance is that educators and other health care professionals need to be alerted that auditory fatigue could be a sign of mild hearing loss, including in children with HIV and AIDS.

In this study, we found conductive hearing loss to be the predominant type of hearing loss, in line with the findings by Shapiro and Novelli (1998), Matas et al. (2006) and Palacios et al. (2008). Khoza (2002) and De Lange (2007) found different results, where sensorineural hearing loss was dominant. However, both studies were conducted in the adult population. Robbert et al. (2017) found that conductive hearing loss was predominant in children with HIV. Meuller and Pizzo (1997) attributed the high prevalence of hearing loss in children with HIV to an immature immune system, resulting in a high susceptibility to otologic infections and hearing loss, thus amplifying the need for early identification and management. Children with severe immunosuppression are likely to have otologic conditions that will affect their hearing (Feagans et al., 1994). Palacios et al. (2008) reported that audiological abnormalities were more frequent in patients with more prolonged HIV-1 infections, higher viral loads or lower absolute CD4+ cell counts. This phenomenon was not part of the study scope, as the focus was on determining the audiological profile. However, in the South African context, Khoza-Shangase (2010) and De Lange (2007) had different results, where sensorineural hearing loss was dominant.

In this study, it was observed that conductive hearing loss was the most predominant type of hearing loss, and a rising audiometric configuration was predominant, which is in line with conductive hearing loss that could be associated with otitis media (Most & Tsach, 2010). Miziara, Webber, Cuhna, Filho and Neto (2007) conducted a study where they assessed the prevalence of otitis media associated with the use of highly active antiretroviral therapy (HAART) in HIV-positive children. The results revealed that otitis media had a prevalence of 31% and, therefore, the use of HAART resulted in a lower prevalence of otitis media. The age group studied in Miziara et al. (2007) is similar to the current study echoing similar findings. Palacios et al. (2008) correlated a lower CD4 cell count to auditory pathology and concluded that immunocompromised children are susceptible to otologic infections including otitis media. Besides, the researcher speculates that conductive hearing loss could also be related to the time of data collection, during the month of June, when it is winter in South Africa, a season associated with a higher prevalence of upper respiratory tract infections associated with otitis media (Khoza-Shangase, 2011).

Of the 16 (57%) participants with a hearing loss, four (25%) participants presented with unilateral hearing loss whilst 12 (75%) participants presented with bilateral hearing loss. Caregivers suspected hearing loss in 13 (43%) participants, and 24 (80%) participants had never had an audiological assessment even though they had reported ear pathology to other health professionals, indicating a need for caregiver reports of ear-related concerns to be addressed through audiological assessments (Carney & Moeller, 1998; North-Matthiassen & Singh, 2006; Olyer, Oyler, & Matkin, 1988; Paluski & Kaderavek, 2002). The caregivers in this study reported hearing-related concerns and suspicions of hearing loss to other health professionals but no audiological referrals were made for further investigations. Olusanya, Okolo and Ijaduol (2000) reported that the lack of understanding of the impact of hearing loss on academic potential was a contributing factor to such a high incidence of unreported hearing loss in Africa. The lack of the necessary audiological referrals raises a concern regarding the prioritisation of hearing loss in the public health care system, especially to children who are vulnerable to hearing loss (Taha et al., 2010).

Furthermore, in this study, half of the participants reported that their children had repeated a grade, indicating that the presence of a hearing loss had possibly negatively affected scholastic performance. Therefore, there appears to be a possible link in children between immunocompromise and susceptibility to hearing difficulties that negatively impact on academic performance (Most & Tsach, 2010). There are other neurological factors such as the neurotrophic nature of the virus that needs to be considered in the population of children infected with HIV (Gross, Wolf, Elidan, & Eliashar, 2007).

The human immunodeficiency virus is reported to affect the inner ear, as the virus has been reported to be neurotropic and can present with neurological symptoms (Lasis, 2005). Viral agents have been reported to attack the spiral ganglion and acoustic section of the eighth cranial nerve, resulting in the sudden onset of a permanent hearing loss (Gross et al., 2007). In this study, it was observed that participants who have epilepsy and HIV encephalopathy showed abnormal ABR results (Govender, Eley, Walker, Petersen and Wilmshurst, 2014). In addition, it was initially believed that the virus affects only the immune cells of the body, and further research has indicated that the virus attacks and destroys the central nervous system (CNS), with an estimated 10% – 30% of people infected with HIV displaying neurological abnormalities (Khoza-Shangase, 2010; Makar et al., 2012; Harris, et al., 2012). Paken (2007) confirmed that there are neurological changes in normal-hearing adults with HIV and AIDS; however, similar findings in children need further investigation. Hence, a neurological ABR was recommended for inclusion in the test battery for this study to assess neural integrity. The neurological abnormalities can also be associated with hearing-related problems, with reports of auditory processing deficits, central hearing loss and auditory brainstem disorders, which negatively affect language development and communication (Makar et al., 2012). This study supports the principle that ABR is sensitive to detect neurological subtleties, which might not be detected with other tests (Gross et al., 2007). Therefore, immunocompromised children could require audiological assessments, including neurological ABR, for earlier detection of the subtle neurological changes that can affect hearing.

## Conclusion

Whilst the present study findings cannot be generalised to the entire South African population because of the small sample size, it has identified that incidence of hearing loss in children with HIV and AIDS appears to be higher than observed internationally. However, this finding is interpreted with caution because of study limitations. This study supports Khoza-Shangase’s (2011b) the assertion that there is a role for the audiologist in the assessment and management of hearing in children with HIV and AIDS. Furthermore, it was observed that audiological referrals were not facilitated in spite of caregiver reports of hearing concerns by health professionals managing the children. Audiologists need to prioritise health promotion in health facilities, including the ARV clinics. Therefore, children with HIV and AIDS, in this study, as a result of compromised immunity, are prone to hearing loss that needs identification, even if they are asymptomatic, to give them a fair opportunity to succeed in life. Studies on larger sample sizes across various sites need to be conducted to track hearing issues in children with HIV and AIDS. In addition, further studies to investigate neurological changes and hearing status could be explored. The pervasive nature of HIV and its effect on a child’s developmental trajectory must be borne in mind by all health care professionals.
